# Myocardial Dysfunction and Shock after Cardiac Arrest

**DOI:** 10.1155/2015/314796

**Published:** 2015-09-02

**Authors:** Jacob C. Jentzer, Meshe D. Chonde, Cameron Dezfulian

**Affiliations:** ^1^Division of Cardiology, UPMC Heart and Vascular Institute, University of Pittsburgh Presbyterian Hospital, 200 Lothrop Street, Pittsburgh, PA 15213, USA; ^2^Department of Critical Care Medicine, University of Pittsburgh Presbyterian Hospital, 200 Lothrop Street, Pittsburgh, PA 15213, USA; ^3^Safar Center for Resuscitation Research, University of Pittsburgh Presbyterian Hospital, 200 Lothrop Street, Pittsburgh, PA 15213, USA

## Abstract

Postarrest myocardial dysfunction includes the development of low cardiac output or ventricular systolic or diastolic dysfunction after cardiac arrest. Impaired left ventricular systolic function is reported in nearly two-thirds of patients resuscitated after cardiac arrest. Hypotension and shock requiring vasopressor support are similarly common after cardiac arrest. Whereas shock requiring vasopressor support is consistently associated with an adverse outcome after cardiac arrest, the association between myocardial dysfunction and outcomes is less clear. Myocardial dysfunction and shock after cardiac arrest develop as the result of preexisting cardiac pathology with multiple superimposed insults from resuscitation. The pathophysiology involves cardiovascular ischemia/reperfusion injury and cardiovascular toxicity from excessive levels of inflammatory cytokine activation and catecholamines, among other contributing factors. Similar mechanisms occur in myocardial dysfunction after cardiopulmonary bypass, in sepsis, and in stress-induced cardiomyopathy. Hemodynamic stabilization after resuscitation from cardiac arrest involves restoration of preload, vasopressors to support arterial pressure, and inotropic support if needed to reverse the effects of myocardial dysfunction and improve systemic perfusion. Further research is needed to define the role of postarrest myocardial dysfunction on cardiac arrest outcomes and identify therapeutic strategies.

## 1. Introduction

Cardiac arrest (CA) is a leading cause of death in the United States, affecting more than half a million Americans each year [1–4]. Survival rates after CA remain poor even after achieving return of spontaneous circulation (ROSC), and approximately 60% of patients admitted to the hospital after CA die from complications [1–4]. Deaths within the first 24 hours after ROSC typically result from refractory shock producing recurrent CA or multiorgan system failure (MOSF), while later deaths result from neurological injury [5–7]. Most deaths after in-hospital CA (IHCA) result from refractory shock, recurrent CA, and MOSF, while most deaths after out-of-hospital CA (OHCA) result from neurological injury [5–8]. Postcardiac arrest syndrome (PCAS) refers to the constellation of abnormalities that develops after resuscitation from CA, including neurological dysfunction, postarrest myocardial dysfunction (PAMD), systemic ischemic/reperfusion injury (IRI), and persistent precipitating pathology [9, 10]. PAMD results from acute cardiac injury from CA resuscitation superimposed on the acute or chronic cardiac condition that caused CA. Mechanisms of PAMD overlap with those producing cardiac dysfunction during myocardial infarction (MI), sepsis, and stress-induced cardiomyopathy and after cardiopulmonary bypass (CPB). Hemodynamic instability and shock after CA may result from PAMD and/or from systemic vasodilation from systemic inflammatory response syndrome (SIRS) [11–14]. In this review, we will discuss the epidemiology, pathophysiology, and management of PAMD and shock after ROSC.

## 2. Epidemiology of PAMD and Shock after ROSC

The true incidence of PAMD after CA in humans remains uncertain due to the small sample sizes, variable definitions, and inconsistent cardiac function assessment in published studies (Table 1) [8, 14–17]. Manifestations of PAMD include low cardiac index (CI), left ventricular systolic dysfunction (LVSD), left ventricular (LV) diastolic dysfunction, and/or right ventricular dysfunction. Echocardiography is the first-line diagnostic test for PAMD, and reduced left ventricular ejection fraction (LVEF) is the most commonly reported manifestation of PAMD. Human studies suggest that two-thirds of patients resuscitated from CA have LVSD within the first 24 hours after ROSC, with a mean LVEF of approximately 40% ± 5% (Table 1) [8, 14–21]. Shock and vasopressor dependence after ROSC are not surrogates for PAMD because they may result from vascular dysfunction without PAMD [14]. PAMD does not reliably predict vasopressor requirements and has not been consistently linked with adverse outcomes when corrected for severity of CA and presence of shock and vasopressor support. It remains uncertain whether PAMD directly impairs survival and recovery after CA or whether development of PAMD merely reflects a greater degree of ischemic injury sustained during severe CA. Rearrest early after ROSC appears to occur in at least 6% of transported post-ROSC patients [22]. As myocardial dysfunction predisposes to sudden death, it is likely that a portion of early post-ROSC rearrests and deaths result directly from underlying PAMD [8].

### 2.1. Low Cardiac Output after CA

In 2002, Laurent et al. reported hemodynamic data in 165 OHCA survivors who underwent systematic coronary angiography [14]. Hemodynamic instability requiring pulmonary artery catheter (PAC) placement and vasopressor support occurred in 55% of patients, predicted by a higher cumulative epinephrine dose and number of countershocks during cardiopulmonary resuscitation (CPR). Hypotension with a low CI (mean 2 L/min/m^2^) developed 6–8 hours after intensive care unit arrival despite aggressive fluid resuscitation (median 8 liters over 72 hours) for low cardiac filling pressures. Vasopressor requirements peaked at 24 hours, with a progressive increase in CI and a reduction in systemic vascular resistance (SVR) leading to persistent vasopressor requirements for up to 72 hours despite normalization of CI. Persistently low CI at 24 hours was associated with early death due to MOSF, but the surviving patients had restoration of normal hemodynamics by 72 hours. Mean LVEF at coronary angiography was lower in patients with hemodynamic instability (32% versus 43%), although only half of these patients had an acute coronary occlusion. Neurologic outcomes did not differ based on the presence or absence of hemodynamic instability.

Oksanen et al. reported on 47 patients who underwent PAC placement during therapeutic hypothermia (TH) after resuscitation from VF OHCA [23]. A low CI (<1.5 L/min/m^2^) developed in 66% during the first 12 hours after ROSC, with nadir CI values at 6 hours; the remaining patients without apparent PAMD had mean CI in the 1.5–2 L/min/m^2^ range. Low CI resulted from reduced stroke volume (SV) index and low heart rate (HR) that responded to low-dose dobutamine. There were no clinical, laboratory, or hemodynamic predictors of low CI, and low CI did not predict clinical adverse outcomes. Trzeciak et al. reported on a highly selected subset of 333 CA survivors undergoing invasive hemodynamic assessment with a PAC from a registry of 8736 total CA patients [24]. The initial CI was below 2.5 L/min/m^2^ in 49% of these patients and below 2.0 L/min/m^2^ in 28%; low CI was not a risk factor for adverse outcomes, although the requirement for inotropic support did increase mortality. A significant limitation of these studies is the selective monitoring of CI.

### 2.2. Abnormal Systolic Function

PAMD was first described in swine as decreased LVEF (from 55% to 20%) and increased LV end diastolic pressure within 30 min of ROSC that recovered to baseline within 48 hours [25, 26]. In 2005, Ruiz-Bailén et al. reported on serial echocardiography in 29 CA survivors without cardiac etiology or prior cardiac disease [15]. At 24 hours, an LVEF <55% was identified in 69% of patients, with a mean LVEF of 28% in these patients with PAMD and a mean LVEF of 42% overall. LVEF at 24 hours was higher in survivors than in nonsurvivors (38% versus 22%), but there were no significant predictors of reduced LVEF at 24 hours. Echocardiographic LVEF increased each week with normalization over the first month in survivors; nonsurvivors who underwent serial echocardiography did not have an improvement in LVEF. Apical segments displayed more severe wall motion abnormalities (WMA) with sparing of basal segments, a finding also seen in stress cardiomyopathy [27].

Preexisting LVSD cannot be reliably distinguished from reversible PAMD as the cause of reduced LVEF after ROSC in CA survivors without acute MI and may be more prognostically important. In 2008, Gonzalez et al. reported on 613 patients who had an echocardiogram within 3 months prior to IHCA [8]. LVEF decreased by one-quarter from its baseline value (from 43% prior to IHCA to 32% after IHCA) in the 84 patients who had an echocardiogram within 72 hours after IHCA, with a similar relative reduction in LVEF regardless of prearrest LVEF. Prearrest LVEF <45% was a predictor of lower survival after IHCA, and patients with LVSD prior to IHCA were more likely to die of refractory shock after ROSC.

In 2012, Dumas et al. reported on 422 OHCA survivors without obvious noncardiac arrest etiology who underwent early coronary angiography [16]. A reduced LVEF <40% was present in 34% of patients at the time of coronary angiography, including 17% of patients with recent coronary occlusion and 36% of patients without. Gaieski et al. performed echocardiography in 15 patients within 6 hours after OHCA, revealing a mean LVEF of 39% that improved to 43% at 72 hours in the 10 survivors who underwent repeat echocardiography [18]. Ameloot et al. reported a mean LVEF of 42% in 82 patients after ROSC, with a lower mean LVEF of 34% in the subgroup of patients with low S_cv_O_2_ ≤66% that correlated with a lower mean cardiac output (CO) of 3.2 L/min [19].

The most comprehensive study of PAMD comes from a subset of 171 patients enrolled in the Targeted Temperature Management (TTM) study comparing 36°C versus 33°C who underwent serial echocardiography and PAC placement [20]. Mean LVEF was 35–39% upon ICU admission and increased slightly to 39–42% (mean 4% increase) by 48 hours, with a greater increase in the 36°C group. The peak systolic myocardial tissue Doppler (*s*′) velocity and tricuspid annular plane systolic excursion (TAPSE) values were reduced on admission and increased by 48 hours. The CI was lower in the 33°C group despite similar vasopressor requirements, LVEF, TAPSE, and *s*′ values, primarily due to reduced HR with a lesser reduction in SV and similar mean arterial pressure (MAP) due to higher SVR. In the overall TTM study, LVEF on the first day was severely reduced (<30%) in 28% of patients and moderately reduced (30–50%) in 48%, with normal LVEF (>50%) in only 25% [21]. LVEF distribution did not differ between patients with higher and lower vasopressor requirements or between target temperature groups, emphasizing the dissociation between LVSD and systemic hemodynamics.

### 2.3. Abnormal Diastolic Function

Profound diastolic dysfunction was first demonstrated in animal models of PAMD prior to its description in humans [26, 28]. In 2007, Chang et al. performed echocardiography at 6 hours after ROSC in 58 OHCA survivors, reporting LVEF as a measure of LV systolic function and isovolumetric relaxation time (IVRT) as a measure of LV diastolic function [17]. Prior MI and higher epinephrine doses were associated with lower LVEF, and LVEF below 40% was associated with worse survival and lower rates of neurological recovery on univariate but not multivariate analysis. A prolonged IVRT ≥100 ms (reflecting diastolic dysfunction) was associated with noncardiac etiology of arrest and nonshockable arrest rhythm and remained an independent predictor of poor survival after adjustment for age, initial cardiac rhythm, epinephrine dose, and CPR duration. In the study by Bro-Jeppesen et al., early mitral annular diastolic tissue Doppler (*e*′) velocity was reduced immediately after ROSC and increased over the first 48 hours, suggesting transient myocardial diastolic dysfunction mirroring the systolic dysfunction reflected by reduced *s*′ velocities [20].

### 2.4. Hypotension and Shock after ROSC

Arterial hypotension with systolic blood pressure (SBP) <90–100 mmHg or mean arterial pressure (MAP) <60–65 mmHg is present in 47–73% of patients after ROSC, and vasopressor support is required in 52–72% of CA survivors [14, 17, 21, 24, 29–32]. Hypotension, shock, and the need for vasopressor support after ROSC consistently predict worse overall or neurologically intact survival after CA, with an inverse association between MAP and survival [19, 21, 24, 29–35]. Patients who require multiple and/or more potent vasopressors have worse outcomes, and the cardiovascular SOFA score carries the greatest prognostic value of all the SOFA subscores in patients with MOSF after CA [21, 34–36]. Shock after ROSC produces recurrent CA and MOSF and may impair brain perfusion and neurological recovery [37]. Survivors with favorable neurological outcomes have higher MAP and less hypotension than nonsurvivors and patients with poor neurological outcomes, even among patients requiring vasopressor support [19, 24, 31–33]. Hypotension may simply be an overall marker of CA severity, but disrupted cerebral blood flow autoregulation after ROSC may lead to cerebral hypoperfusion during hypotension [38]. Up to 35–80% of patients require inotropic support after ROSC, although rates are highly variable between studies [24, 39–41].

Laurent et al. first demonstrated that shock after CA and ROSC evolves from a low-output state with low CI from PAMD to a vasodilated state with low SVR, combined with a need for significant ongoing fluid resuscitation from abnormal vasodilation and capillary leak from SIRS, mimicking septic shock [14, 23]. Post-ROSC shock often develops after a brief “honeymoon period” lasting up to 6 hours, followed by a low-output state and then worsening vasodilation with increasing vasopressor requirements peaking at 24 hours and gradual resolution over the subsequent 24–48 hours [14, 23]. Higher initial lactate levels predict higher vasopressor doses, suggesting that a greater initial ischemic insult leads to cardiovascular failure [21, 42, 43].

## 3. Pathophysiology of PAMD and Shock after ROSC

**Figure 1:**
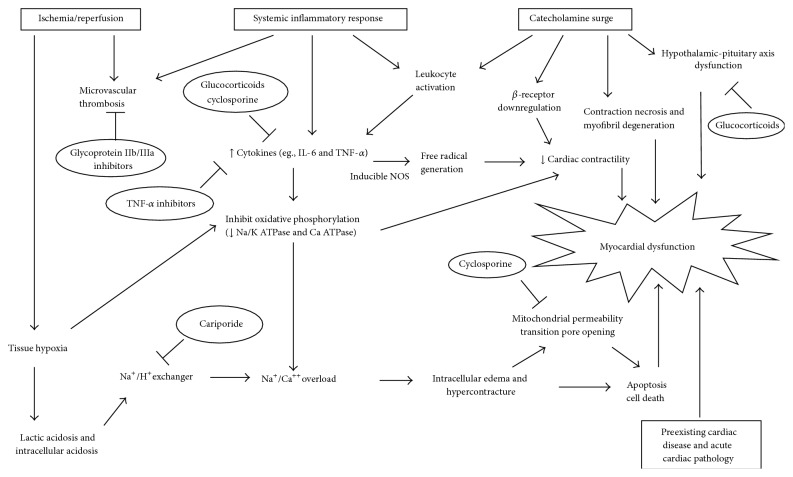
Pathophysiologic mechanisms involved in postarrest myocardial dysfunction. Boxes represent major contributing etiologies. Circles represent therapeutic interventions explored in experimental models of cardiac arrest.

Multiple interacting processes contribute to the reversible deterioration of cardiac function after CA, leading to acute cardiac dysfunction superimposed on underlying structural heart disease (Figure 1). The triggering etiology of CA often produces cardiac dysfunction, but these acute and chronic cardiac conditions are conceptually distinct from true PAMD and are more appropriately considered as precipitating pathology. Three major pathways contribute to PAMD—cardiovascular IRI, catecholamine-induced myocardial injury, and cytokine-mediated cardiovascular dysfunction [44]. PAMD shares pathophysiological and clinical features with three better-characterized conditions, namely, post-CPB myocardial dysfunction, stress-induced cardiomyopathy, and septic cardiomyopathy, respectively [27, 45, 46]. Microvascular dysfunction, adrenal insufficiency, mitochondrial dysfunction, cardiac stunning from direct-current countershocks, and cardiovascular effects of iatrogenic interventions including TH further contribute to PAMD and shock after ROSC [44]. Current management of PAMD and shock is supportive and therapies targeting the underlying pathophysiology have not yet been investigated in clinical studies with patient-centered outcomes. Prevention of PAMD will require interventions targeting multiple pathways in order to produce clinical benefits, and PAMD remains a promising area of postresuscitation research.

### 3.1. Ischemia/Reperfusion Injury

IRI is one of the primary underlying mechanisms linking CA to MOSF, PAMD, and shock [9, 44]. IRI produces myocardial injury during MI and cardiac stunning after CPB via overlapping cellular mechanisms [45, 47]. Unlike focal myocardial ischemia due to MI, the entire myocardium is affected in CA and after CPB, leading to transient but global changes in cardiac systolic and diastolic function. Ischemia produces cellular energy depletion and lactic acidosis from anaerobic metabolism. Cellular energy depletion leads to failure of the membrane Na/K ATPase pump with intracellular sodium overload and cell edema that is worsened by sodium influx through the membrane Na/H exchanger (NHE) due to intracellular acidosis [47, 48]. Intracellular sodium accumulation induces calcium influx through the Na/Ca exchanger, leading to myocardial cellular calcium overload exacerbated by failure of the Ca ATPase due to energy depletion [47, 48]. Intracellular calcium overload produces harmful effects including downstream activation of calcineurin and initiation of cellular apoptosis by opening of the mitochondrial permeability transition pore (MPTP), along with impaired diastolic relaxation and predisposition to arrhythmias [49]. The cellular and hemodynamic effects of cardiovascular IRI overlap with the adverse effects of persistent lactic acidosis [21, 42, 43, 48]. With restoration of blood flow after transient ischemia, overproduction of toxic reactive oxygen species (ROS) leads to a second wave of cellular injury [47]. Profound myocardial cellular energy depletion leads to tetanic cardiac muscle contraction leading to progressive myocardial wall thickening and reduction in cavity volume, a potentially irreversible state called ischemic contracture [50].

Cyclosporine is a calcineurin inhibitor that ameliorates the adverse effects of cellular calcium overload by inhibiting MPTP opening and apoptosis, in addition to anti-inflammatory effects [51, 52]. Cyclosporine prevents IRI in preclinical animal models of PAMD and humans with MI and those undergoing CPB. Preclinical animal models have shown an improvement in PAMD after cyclosporine administration during CA [51–53]. A rat study by Huang et al. showed improved LV systolic function, cardiac output, and mortality when cyclosporine was administered during CA but not when cyclosporine was administered after ROSC [52]. A rabbit study by Cour et al. showed similar improvements in post-ROSC survival and PAMD when cyclosporine was administered at the establishment of reflow [51]. Both studies linked the beneficial effects of cyclosporine to inhibition of MPTP opening [51, 52]. Gill et al. improved cardiac and mitochondrial function in piglets subjected to asphyxial CA who received cyclosporine [53]. Piot et al. demonstrated significant reduction in infarct size in acute MI patients who received cyclosporine compared to placebo, leading to improvements in LV remodeling [54, 55]. Recent studies have shown reductions in myocardial injury with administration of cyclosporine in humans undergoing CPB [56, 57]. These preclinical studies in multiple animal models of CA along with human data in similar disease processes make cyclosporine a promising agent for prevention of PAMD.

The NHE is another potential therapeutic target for prevention of cellular injury during IRI. Multiple animal studies have shown improvements in PAMD and/or mortality with administration of NHE inhibitors (such as cariporide) during CA, including improved hemodynamics and reductions in LVSD and/or arrhythmias [58–64]. Mentzer et al. reported the effects of cariporide in the large EXPEDITION study of patients undergoing CPB, demonstrating a reduction in myocardial injury biomarkers but an increased rate of mortality and cerebrovascular events with cariporide [65]. This human study showing increased neurologic injury with cariporide has reduced enthusiasm for the use of this drug to prevent PAMD, given the importance of neurologic injury for prognosis after CA. Animal studies suggest a beneficial effect of the traditional Chinese medicine Shen-Fu on PAMD via inhibition of IRI and myocardial apoptosis [66, 67].

### 3.2. Inflammatory Cardiovascular Dysfunction

Systemic IRI after ROSC triggers release of inflammatory cytokines leading to SIRS that mimics sepsis, even in the absence of infection [11–13, 68]. The inflammatory response after ROSC is characterized by polymorphonuclear leukocyte activation, adhesion molecule expression, ROS production from inducible nitric oxide synthase (iNOS), and release of cytokines, such as interleukin-6 (IL-6) and tumor necrosis factor-alpha (TNF-*α*) [11, 13, 44, 69, 70]. Like sepsis and the vasoplegia that can occur after CPB, the SIRS that follows ROSC produces pathological vasodilation, depressed cardiac function, and MOSF from direct myocardial depression by cytokines and uncontrolled vasodilation resulting from iNOS activation [12, 46, 71]. Various cytokines have direct depressant effects on cardiac myocyte contractility, contributing to both systolic and diastolic dysfunction in septic cardiomyopathy [46, 72–74]. The intensity of the inflammatory response in both septic shock and postarrest syndrome may explain the high associated mortality in these conditions. Cytokine overproduction also occurs after CPB, and anti-inflammatory therapy with corticosteroids and other agents can reduce myocardial dysfunction after CPB in animal models [71, 75–78]. Corticosteroid treatment in humans undergoing CPB also reduces levels of inflammatory markers and appears to be associated with clinical benefits including reduced need for vasopressors and fewer arrhythmias [71, 78]. Bro-Jeppesen et al. found that IL-6 levels predicted vasopressor requirements and mortality in the TTM trial, confirming the importance of inflammatory mediators in shock after ROSC [13, 68]. Other studies have confirmed the importance of IL-6 levels for predicting MOSF and outcomes after ROSC, with a less consistent association between C-reactive protein levels and adverse outcomes [79, 80]. Therapies targeting cytokine removal have shown some promise for treatment of circulatory dysfunction after CA, suggesting that inflammation may be a modifiable risk factor for death and PAMD [81]. Mitochondrial dysfunction can result from excess cytokine activity as well as the cellular effects of IRI and oxidative stress from elevated ROS, contributing further to myocardial dysfunction via impaired energy metabolism [82, 83]. Mitochondrial dysfunction can impair cellular oxygen utilization, leading to lactic acidosis despite adequate tissue perfusion, a state characterized by high S_v_O_2_ levels and poor prognosis [19, 83].

TNF-*α* is a major mediator of cytokine-induced cardiovascular dysfunction that directly impairs cardiac contractility, beta-adrenergic responsiveness, and mitochondrial function [70, 73, 82, 84]. Biologic inhibitors of TNF-*α*, including infliximab and etanercept, have shown benefit in preclinical animal models of CA. Administration of infliximab during the periarrest period improved cardiovascular function in pigs, as demonstrated by improved MAP, SV, and short term survival [70, 85–87]. Etanercept failed to reproduce the benefits seen with infliximab in the same model [86]. Inhibition of cytokine production may contribute to the improvements in cardiovascular function seen with cyclosporine and corticosteroids after CA in animal models and limited human studies [51–53, 88, 89]. The no-reflow phenomenon, characterized by impaired or absent microvascular perfusion despite restoration of macrovascular flow, can occur in the brain and other organs after resuscitation from CA as it does in the myocardium after reperfusion therapy in acute MI [90, 91]. Endothelial damage from IRI and cytokine activation produces abnormal vascular permeability, coagulation cascade activation, tissue edema, and microvascular occlusion that further impair tissue perfusion [12, 44]. GpIIb/IIIa inhibitors such as abciximab and eptifibatide have improved myocardial microvascular perfusion in preclinical animal models of PAMD, without clear improvements in cardiac function or systemic microcirculatory perfusion [92, 93]. Similarly, improvements in microvascular function with GLP-1 infusion failed to reduce PAMD in pigs [94].

### 3.3. Catecholamine-Induced Cardiac Dysfunction

Catecholamine-mediated cardiotoxicity is another major mechanism contributing to PAMD. Excess levels of catecholamines (particularly epinephrine) can provoke cardiac dysfunction, including stress-induced (takotsubo) cardiomyopathy [27]. High doses of epinephrine (as are administered during CPR) can provoke stress-induced cardiomyopathy in humans [95]. Higher epinephrine doses during CPR predict PAMD in human studies and epinephrine given during resuscitation increases severity of PAMD in animal studies, an effect ameliorated by beta-blockade [14, 17, 96–98]. Catecholamine excess produces myocardial injury and cardiac dysfunction through multiple mechanisms including calcium overload, ROS overproduction, and beta-receptor downregulation and desensitization [27, 99]. Beta-receptor downregulation also occurs in animal models of PAMD in the absence of epinephrine treatment and has also been documented in myocardial dysfunction after CPB [100, 101]. Despite theoretical beta-receptor downregulation in PAMD, most patients respond well to low doses of beta-agonists such as dobutamine [23, 26, 102–104]. The apex of the left ventricle possesses a higher beta-adrenergic receptor concentration, explaining the predisposition to apical hypokinesis seen in stress cardiomyopathy and some studies of human PAMD [15, 27, 99].

Recent observational studies have called the use of epinephrine during CPR into question, showing higher rates of ROSC but lower rates of neurologically intact and overall survival [105]. No difference in mortality was seen with higher epinephrine doses in randomized trials compared to standard dose epinephrine during CPR [106, 107]. Studies using less beta-adrenergic vasopressors such as norepinephrine or vasopressin during CPR likewise have not shown consistent effects on mortality when compared to epinephrine, although certain subgroups appeared more likely to benefit in the case of vasopressin [107, 108]. Effects on myocardial function were not explicitly examined in the majority of these studies, although one study found a potentially harmful effect of epinephrine on post-ROSC hemodynamics with lower CI in patients who had received higher cumulative epinephrine doses during CPR [109]. Reducing epinephrine doses during CPR has the potential to reduce the severity of cardiovascular failure after ROSC.

### 3.4. Relative Vasopressin and Cortisol Deficiency

In two studies, Mentzelopoulos et al. randomized a total of 368 patients suffering IHCA to epinephrine alone or epinephrine with vasopressin and methylprednisolone during CPR, followed by ongoing hydrocortisone therapy or placebo after ROSC [88, 89]. The vasopressin and corticosteroids groups needed less epinephrine during CPR and had higher rates of ROSC and reduced need for vasopressors after ROSC, with improved functional and overall survival as seen in a pilot study [88, 89]. It remains uncertain whether the benefits seen in these studies were due to a harmful effect of epinephrine or a beneficial effect of vasopressin and/or corticosteroids. Prior studies have demonstrated endocrine dysfunction with relative deficiency of vasopressin and cortisol after ROSC, allowing for physiological repletion to have synergistic effects on shock reversal as seen in the studies by Mentzelopoulos [88, 89, 110–114]. Vasoplegia after ROSC may be associated with a relative vasopressin deficiency, as seen in vasodilatory shock from sepsis or after CPB [71, 110, 115]. Low-dose vasopressin has proven effective for shock reversal in all of these vasoplegic states by augmenting adrenergic vasoconstriction and opposing pathological vasodilation, although effects on mortality have been less consistent [71, 115, 116]. Recent animal studies have demonstrated that vasopressin may inhibit downstream receptor second messenger cascades to potentially ameliorate cellular toxicity from excessive beta-adrenergic stimulation [117]. Abnormalities of adrenal function leading to functional adrenal insufficiency appear common after CA, with greater abnormalities identified in nonsurvivors [111–114]. Similar abnormalities of adrenal function are well described in septic shock, and the same low-dose hydrocortisone regimens have proven effective for shock reversal in septic shock and post-ROSC shock [88, 89, 118].

### 3.5. Additional Factors Contributing to PAMD and Shock after ROSC

Several other iatrogenic factors can contribute to cardiovascular dysfunction after CA. The administration of direct-current countershocks during CPR is known to produce myocardial stunning [119]. Animal models have demonstrated that countershocks decrease cardiac contractility, decrease CI, and increase LV end diastolic pressure in a manner dependent on energy and waveform [120, 121]. Human studies show deterioration in hemodynamics and cardiac function after countershocks delivered by implantable defibrillators [122]. Increased number of countershocks is associated with PAMD in some studies, although more countershocks may be a marker of longer CPR duration (as is true for higher cumulative epinephrine dose) [14].

Several medications commonly administered after CA may affect cardiovascular function, including antiarrhythmics and sedatives. Antiarrhythmics such as amiodarone and beta-blockers have negative inotropic effects which may impair systemic hemodynamics in the setting of PAMD. Propofol often produces hypotension from systemic vasodilation and direct myocardial depression and may impair the response to vasopressors and inotropes, particularly in patients with cardiovascular dysfunction [123–126]. Post-ROSC patients receiving propofol and remifentanil had higher rates of hypotension and greater need for vasopressors than patients sedated with midazolam and fentanyl, despite similar outcomes [127]. Adverse hemodynamic effects, particularly vasodilatory hypotension, can be seen with other sedatives and intravenous antiepileptic drugs such as phenytoin and valproic acid. Despite the necessity of vasopressors to maintain systemic hemodynamics in many patients after ROSC, excessive use of these drugs may impair microvascular function and tissue perfusion, in addition to provoking recurrent arrhythmias and potentially increasing the risk of adverse outcomes [36, 128, 129].

TH and TTM have become central to reducing neurological injury and improving outcomes after OHCA [130–133]. Mild TH alters systemic hemodynamics and myocardial performance and has improved PAMD in animal models [134]. The effects of TH on isolated myocardium include increased inotropy and impaired diastolic relaxation, but reduced HR and increased SVR dominate the clinical hemodynamic effects of TH [134]. Bernard et al. demonstrated that patients randomized to TH had significantly lower CI, higher SVR, and lower HR during the first 12 hours after ROSC without a significant difference in MAP or SV [130]. In this study, patients receiving TH had improved clinical outcomes, suggesting that hemodynamic changes resulting from TH are not harmful per se. Observational studies have shown similar vasopressor requirements in patients receiving TH versus normothermia, with persistence of vasopressor dependence after rewarming in patients receiving TH [36, 41, 135]. On the contrary, patients in the TTM trial randomized to 33°C had increased vasopressor requirements compared to the 36°C group despite similar MAP [21]. In addition, patients with shock in the 33°C group of the TTM trial had higher lactate levels and a trend to worse outcomes when adjusted for baseline characteristics [37]. This supports the use of TTM to 36°C in patients after ROSC independent of the presence of shock or vasopressor dependence and suggests caution when using mild TH to 33°C in patients with shock after ROSC. Interestingly, small studies of overt cardiogenic shock (including patients after CA) have shown improvements in hemodynamics after induction of mild TH, without apparent adverse effects [136–138].

## 4. Therapeutic Approach to PAMD and Shock after ROSC

There are no randomized, controlled clinical trials examining different treatment approaches or interventions for PAMD and shock after CA. Early goal-directed therapy (EGDT) has been advocated for hemodynamic optimization of shock after CA based on similarities to septic shock, although recent sepsis studies have failed to show that EGDT improves outcomes [9, 10, 18, 39, 40, 139]. Observational studies show reduced mortality after instituting EGDT protocols in post-CA patients as part of a multifactorial quality improvement strategy including TH and routine coronary angiography [18, 39, 40]. It is difficult to draw conclusions regarding the effects of the EGDT protocol itself on outcomes in the context of these complex interventions.

### 4.1. Optimizing Preload

**Figure 2:**
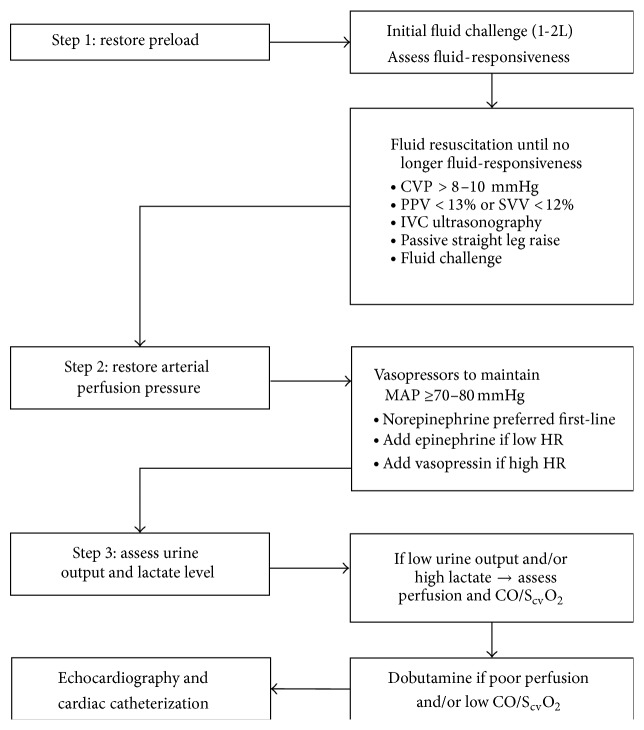
Suggested early goal-directed hemodynamic optimization strategy for patients with hypotension or hypoperfusion after return of spontaneous circulation following cardiac arrest. CVP, central venous pressure; PPV, pulse pressure variation; SVV, stroke volume variation; IVC, inferior vena cava; MAP, mean arterial pressure; HR, heart rate; S_cv_O_2_, central venous oxygen saturation; CO, cardiac output.

Restoration of adequate preload is the first step in resuscitation of patients with hypotension, shock, or low CO after ROSC (Figure 2) [10]. Large volumes of fluid may be required to maintain adequate CO due to systemic capillary leak from systemic IRI and cytokine release [14]. Initial resuscitation with 1-2 L of isotonic crystalloid is recommended in hypotensive patients after ROSC [10]. Early aggressive fluid resuscitation targeting hemodynamic goals may reduce overall fluid requirements. A central venous pressure of 8–12 mmHg is recommended by guidelines and has been used as a fluid resuscitation endpoint in most studies of EGDT after CA [9, 10, 18, 20, 39]. Our institutional protocol involves use of dynamic measures such as pulse pressure and stroke volume variation to assess fluid-responsiveness due to the limitations of central venous pressure as a measure of preload, particularly in the setting of cardiac dysfunction. Diastolic dysfunction after ROSC predisposes patients to both inadequate CO during relative hypovolemia and pulmonary edema from aggressive fluid administration [140].

### 4.2. Restoring Arterial Pressure

Vasopressor support can counteract the pathologic vasodilation resulting from vascular IRI and inflammatory cytokine release after ROSC, although no randomized studies have explicitly studied specific vasopressors after CA [141]. The need for vasopressors to restore MAP and support tissue perfusion (Figure 2) often lasts for approximately 48–72 hours, even after CO normalizes [14]. Arterial pressure monitoring is prudent for hemodynamically unstable patients with PAMD or shock requiring vasopressor support. Use of norepinephrine as a first-line vasopressor is supported by studies showing favorable outcomes with lower risk of arrhythmias in heterogeneous shock patients receiving norepinephrine [129, 142]. Dopamine is a suboptimal vasopressor based on its lower efficacy and increased risk of tachyarrhythmias and mortality in cardiogenic shock patients in the SOAP-II study [129]. Our institutional protocol is to add epinephrine as the second-line vasopressor for patients with refractory shock, low CO, and/or bradycardia. The optimal HR for patients after CA remains unknown, and many patients tolerate bradycardia remarkably well if they can maintain CO by increasing SV, especially in the presence of diastolic dysfunction or during hypothermia. Vasopressin can be added to counteract refractory vasoplegia in patients with preserved CO and/or tachycardia and may be useful in patients with recurrent tachyarrhythmias due to its lack of proarrhythmic effects [116]. Low-dose hydrocortisone can be added for patients not responding promptly to standard vasopressor therapy and has proven efficacy for reversal of refractory vasodilatory shock [88, 89, 118]. In addition to relative adrenal insufficiency, ionized hypocalcemia and lactic acidosis with severe acidemia are frequent contributors to refractory shock after ROSC [21, 43, 111–114, 143].

The optimal MAP for patients after ROSC remains uncertain, with no consistency between published protocols for hemodynamic support after ROSC. Current American Heart Association guidelines recommend maintaining systolic BP goal ≥90 mmHg and MAP ≥65 mmHg [9, 10]. A MAP ≥70 mmHg is associated with better outcomes after CA, while a MAP <65 mmHg has been associated with poor outcomes and impaired cerebral oximetry [19, 21, 32]. One study reported maximal survival in patients with a MAP range of 76–86 mmHg and maximal cerebral oximetry values with a higher MAP range of 87–101 mmHg [19]. Several authors have recommended a MAP goal ≥80 mmHg to prevent cerebral hypoperfusion in the presence of impaired cerebral blood flow autoregulation after CA [18, 32, 38]. However, elevating the MAP from 70 mmHg to 90 mmHg using norepinephrine failed to improve cerebral oximetry after CA in a small study [144]. Our institutional protocol is to maintain MAP ≥80 mmHg after ROSC, except in patients with severe shock requiring high doses of vasopressor agents when a lower goal of ≥65–70 mmHg is used to avoid excessive vasopressor doses. Vasodilator and/or beta-blocker therapy to maintain MAP ≤100 mmHg is reasonable to reduce myocardial afterload and oxygen demand in patients who remain hypertensive after adequate sedation [9]. One study showed worse outcomes and poorer cerebral oximetry in patients with MAP >100 mmHg after ROSC [19].

### 4.3. Supporting Tissue Perfusion

Inotropic support may be required to treat persistently low CO after fluid resuscitation (Figure 2), potentially warranting PAC insertion [141]. Indications for inotropic support for shock after CA remain uncertain, although EGDT protocols often recommend inotropic support to augment low CO and/or low S_cv_O_2_ [18, 39]. Inotropic agents can aggravate tachyarrhythmias or myocardial ischemia, and no CO value is optimal for all patients [134]. Inotropic agents should be reserved for patients with impaired end-organ perfusion in addition to inadequate CO and/or systemic oxygen delivery, that is, low urine output and/or persistent lactic acidosis in the presence of a low CO or S_v_O_2_. Artificially augmenting CO with inotropic support based on low S_cv_O_2_ is unlikely to be beneficial in the absence of impaired end-organ perfusion [134]. Reasonable therapeutic goals for inotropic support include a urine output ≥0.5–1 mL/kg/h (up to 1.5 mL/kg/h during TH) and S_cv_O_2_ ≥70% with a declining or normal lactate [9, 10]. One study found higher survival in patients with a S_v_O_2_ of 67–72%, with optimal cerebral oximetry at S_v_O_2_ values of 70–75%; elevated S_v_O_2_ values >75% may suggest failure of end-organ oxygen utilization due to mitochondrial dysfunction or microvascular shunting, with an adverse prognosis [19]. Dobutamine doses of 2–5 mcg/kg/min are usually effective for augmenting CO, with no added efficacy and more adverse effects at doses >10 mcg/kg/min [10, 23, 26, 102–104]. The vasodilatory properties of dobutamine may be useful for improving splanchnic perfusion in patients requiring vasopressors [145]. Low-dose dopamine or epinephrine can augment CO and HR in hypotensive patients while avoiding the vasodilatory effects of dobutamine that can exacerbate hypotension when SVR is low [10]. Milrinone carries a higher risk of vasodilatory hypotension but retains efficacy despite beta-receptor downregulation and is less likely to provoke tachyarrhythmias or increase myocardial oxygen demand in selected patients [146].

Patients who have suffered CA due to massive acute MI may develop refractory cardiogenic shock, with a very high mortality rate despite medical therapy [147]. In selected patients, mechanical circulatory support can restore hemodynamic stability and end-organ perfusion [148]. The intra-aortic balloon pump (IABP) appears to provide relatively minimal augmentation of MAP and CO [148]. The large IABP-SHOCK-II trial failed to show a mortality benefit from the use of IABP in revascularized patients with cardiogenic shock after MI [147]. These findings likely apply to patients with PAMD and shock after CA due to MI, because 45% of enrolled patients had been resuscitated from CA. Animal studies suggest greater efficacy of dobutamine than IABP for augmenting hemodynamics after ROSC [104]. The Impella percutaneous left ventricular assist device may be an alternative to IABP after ROSC that provides more robust hemodynamic support [149]. Venoarterial extracorporeal membrane oxygenator (ECMO) pumps have been used as rescue therapy for refractory CA or severe cardiogenic shock after ROSC, and preliminary data suggest that appropriately selected patients can be stabilized on ECMO and survive despite shock refractory to maximal medical therapy [150].

## 5. Conclusion

PAMD is a multifactorial syndrome developing from the interaction between prearrest cardiac pathology and intra-arrest cardiac insults. PAMD has been reported in up to two-thirds of patients resuscitated from CA, even in the absence of prior cardiac disease. Systolic dysfunction of variable severity is commonly identified, with diastolic dysfunction less frequently reported. PAMD may lead to impaired CO requiring vasoactive support, but shock after ROSC is typically dominated by pathologic vasodilation which persists after normalization of CO. The adverse prognostic value of shock and vasopressor dependency after ROSC is clear, although the contribution of PAMD to adverse outcomes remains uncertain. The pathophysiology of PAMD overlaps with myocardial dysfunction developing as a result of IRI seen after CPB, cytokine excess seen in sepsis, and catecholamine toxicity as in stress-induced cardiomyopathy. Echocardiography is the primary tool for diagnosing PAMD, with invasive hemodynamic monitoring typically warranted for patients with PAMD or shock after ROSC. Treatment of PAMD is similar to other forms of shock, including optimization of preload, restoration of perfusion pressure, and augmentation of contractility to ensure tissue perfusion. Future research is needed to explore the independent relationship between PAMD and outcomes after CA, in addition to the optimal approach to management.

## Figures and Tables

**Table 1:** Incidence of left ventricular systolic dysfunction in adult survivors of cardiac arrest. LVEF = left ventricular ejection fraction, LVSD = left ventricular systolic dysfunction (LVEF < 50–60%), and NR = not reported.

Study	Year	Number of patients	% LVSD	Mean LVEF
Laurent et al. [14]	2002	148	NR	37.6%
Ruiz-Bailén et al. [15]	2005	29	69%	42%
Chang et al. [17]	2007	58	NR	53.7%
Gonzalez et al. [8]	2008	84	NR	32%
Gaieski et al. [18]	2009	22	NR	36.9%
Dumas et al. [16]	2012	308	72%	NR
Bro-Jeppesen et al. [20]	2014	154	NR	37%
Bro-Jeppesen et al. [21]	2015	523	75%	NR
Ameloot et al. [19]	2015	82	NR	42%
